# Chronic Pharyngitis

**Published:** 1908-12-05

**Authors:** 


### Laryngology and Rhinology.

### CHRONIC PHARYNGITIS.

Chronic pharyngitis is one of the minor ailments which are apt to receive somewhat scant consideration from the physician, but which may cause to the patient an immense amount of discomfort or even misery. This is especially the case in this condition because the symptoms complained of are not only rather vague but are often out of all proportion to the objective appearances; indeed, the typical granules, the enlarged vessels, and the diffuse congestion are frequently seen in people who do not complain of any discomfort about the throat. It is necessary to insist strongly on this disproportion between the symptoms and the objective signs of pharyngitis; the former must chiefly be relied on in making the diagnosis and in estimating the progress under treatment.

### Varieties and Symptoms.

Three varieties of the affection may be distinguished and are usually described: (1) simple catarrhal pharyngitis; (2) granular pharyngitis; (3) pharyngitis sicca. The ætiology, the symptoms and, except for the granules, the appearances of the first two are identical and they may well be considered together.

As already indicated, the degree of disturbance to be seen is very variable; some patients complain of great discomfort in whom little abnormal can be found, while other people with marked granular hypertrophy suffer no inconvenience. In a marked and typical case the mucous membrane of the pharynx, fauces, and soft palate is congested, and enlarged veins are seen to ramify on the posterior pharyngeal wall; on the latter there is often a thin layer of tenacious mucus which may not be visible until it is wrinkled by touching it with a probe or mop. The uvula is frequently elongated, and is usually relaxed so that it does not retract on gagging or singing a high note. The mucous membrane sometimes appears generally thickened, at other times it looks raw and excoriated. The nodules characteristic of the variety known as "granular pharyngitis" are smooth, slightly raised patches of a pink colour and oval shape measuring about $\frac{1}{8}$ to $\frac{3}{16}$ in. in length; they are caused by a proliferation of the lymphoid nodules about the mouths of the mucous glands, resemble the Peyer's patches of the small intestine, and are chiefly to be seen on the posterior pharyngeal wall. In some cases the mucous thickening occurs principally at the side of the pharynx, just behind the posterior pillars of the fauces, forming the so-called "lateral bands."

In cases of *pharyngitis sicca*, the mucous membrane is thin and smooth and has a glazed appearance as though varnished; its colour is noticeably uniform all over and is generally a dull rather deep red, though it may be pale and anæmic.

The symptoms are generally vague; a feeling of a lump, of tickling, or as if a hair were caught in the throat, are frequent symptoms. There is no pain on swallowing, indeed the patient usually feels more comfortable after a meal as also after painting the throat with any stimulating application. Sometimes dryness of the throat is complained of even when the case is one of hypertrophic, not of dry, pharnygitis. A frequent short ineffective cough is common, but there may be much hawking and expectoration of mucus, especially when the nasopharynx is involved. Although the symptoms are somewhat vague, the degree of discomfort may be extreme.

### Ætiology.

The causes of chronic pharyngitis are very diverse, but they may mostly be ultimately ascribed to irritation either directly or through the blood. Mouth-breathing is a fruitful cause, as also are all forms of nasal catarrh and suppuration. Adenoids and tonsils which have shrivelled in adolescence, are often present; hypertrophied lymphoid nodules are often seen on the pharynx in young people with adenoids and tonsils, but they rarely cause symptoms until later in life. Working in a stuffy or dusty atmosphere is another form of direct irritation, as also is the over-use of tobacco. Indirect causes are anæmia, rheumatism, gout, and, above all, dyspepsia; it would seem that poisons may circulate in the blood which have a selective action on the pharynx in the same way as atropine or muscarin. Carious teeth may act as a source of direct and indirect irritation, as also may over-indulgence in alcohol; the latter is a most important and frequent cause of pharyngitis. Finally, chronic pharyngitis is often seen in those who use the voice much with incorrect production; it forms a large part of the affection known as "clergyman's sore throat" and appears to be due to the congestion brought about by improper use of the vocal organs. There is often a strong neurotic element, which explains the disproportion between the symptoms and the visible signs of disease.

### Treatment.

Any treatment which aims at a permanent cure must include treatment of the cause, and the affection remains notably difficult to treat because it is often but a symptom of chronic dyspepsia or the result of a sedentary life, of over-eating and drinking. Treatment of the constitutional causes cannot be discussed fully here, but it may be said that, roughly speaking, the affection occurs among anæmic women and in plethoric men, and that the two classes require completely different lines of treatment.

As regards local treatment, any stimulating medicament applied to the throat with a brush will give relief. Mandl's solution is much used: Iodine gr. vj., potassium iodide gr. xij., menthol gr. iij., glycerine ℥j.; or perchloride or sulphate of iron may be employed in strengths of one to two drachms to the ounce; a ten per cent. solution of resorcin in glycerine is sometimes very valuable. The use of astringent lozenges is disappointing, and gargles are useless. If naso-pharyngeal catarrh is present, a simple alkaline nasal lotion should be ordered. The judicious use of the galvano-cautery is the most valuable method of local treatment; a broad flat cautery should be used at a dull red heat after thorough cocainisation. Any enlarged lymphoid granules should be destroyed, but the treatment is also useful when no granules are present. Enlarged veins should be obliterated at one or more points, and hypertrophied lateral bands must receive attention. Several sittings are often required. Considerable soreness results, and hot and spiced food must be avoided for a few days. The method is useless or even harmful in cases of pharyngitis sicca.

